# The influence of random microstructure on wave propagation through heterogeneous media

**DOI:** 10.1007/s10704-016-0170-2

**Published:** 2016-11-28

**Authors:** Yilang Song, Inna M. Gitman, William J. Parnell, Harm Askes

**Affiliations:** 1 Department of Mechanical Engineering, University of Sheffield, Sheffield, UK; 2 School of Mathematics, University of Manchester, Manchester, UK; 3 Department of Civil and Structural Engineering, University Sheffield, Sheffield, UK

**Keywords:** Stop-band, Wave filter, Composite material, Laminate, Randomness, Wave propagation

## Abstract

In this paper the influence of mechanical and geometrical properties, both deterministic and stochastic in nature, of a heterogeneous periodic composite material on wave propagation has been analysed in terms of the occurrence of stop-bands. Numerical analyses have been used to identify those parameters that have the most significant effect on the wave filtering properties of the medium. A striking conclusion is that randomness in geometrical properties has a much larger effect than randomness in mechanical properties.

## Introduction

It is well known that heterogeneous materials behave very differently compared to their homogeneous counterparts, in particular when they are subjected to dynamic loading. This is principally ascribed to the presence of wave dispersion in heterogeneous materials, which leads to a wide variety of interesting dynamic effects. One particular well-studied phenomenon caused by wave dispersion is the presence of so-called *stop-bands* or *band-gaps*, i.e. intervals of frequencies where wave propagation does not occur. Well described by Brillouin (1946), the phenomenon has mainly been studied in two-phase materials with periodic structure (Kushwaha et al. 1993; Sigalas and Economou 1994; Vasseur et al. 1994). However, when heterogeneous materials do *not* have a periodic structure, the notion of propagation of waves within the medium is more difficult to quantify precisely.

**Fig. 1:**
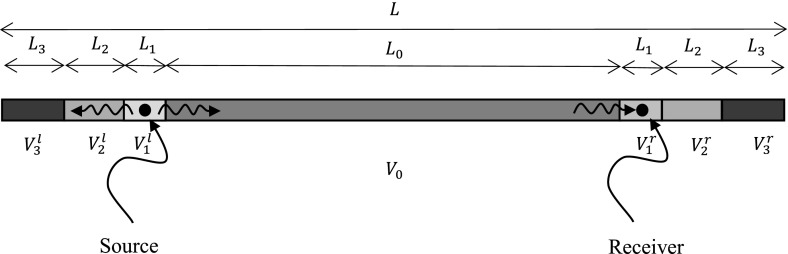
Illustration of the configuration used in numerical simulations

In a material with disorder there is no longer the clear notion of a *stop-band* as in the periodic case (Sheng 1995). The literature discusses *strong* and *weak* disorder, most commonly in the positional disorder of e.g. inclusions in a matrix medium. The transition from weak to strong disorder results in the loss of the band-gap structure present in periodic media. The effective wavenumber becomes complex at all frequencies and so for an infinite medium the theory predicts broadband attenuation, although this depends on the relative magnitude of the imaginary and real parts of the effective wavenumber. Furthermore, in reality the priority is to understand wave propagation through media of *finite* extent and so what is perhaps most important is the magnitude of a transmission coefficient across the medium, measuring the amount of energy that has passed through the system. The aim of this work is therefore to understand the influence of non-periodic internal structure of materials on time-harmonic elastic wave propagation, and specifically how this affects the presence or otherwise of stop-bands.

Analogous to the term *photonic* media associated with electromagnetic waves, heterogeneous *elastic* media composed of periodic arrays of inclusions embedded in a matrix are usually called *phononic* crystals. In some ways this is unfortunate terminology since more recently the study of *heat* transmission in periodic media has also taken place (see e.g. Maldovan 2013) which really *should* be classified as phononic interactions. As described above, the propagation of sound and vibrations in periodic media can be strictly prohibited in certain frequency ranges (Kushwaha and Djafari 1998; Vasseur et al. 1994, 2002). Hence, it is possible to use phononic crystals in order to design elastic wave filters to create silent environments, amongst other applications. Understanding the stop-band phenomenon aids more effective design of materials by enabling better control of wave propagation through them. Theoretically, several methods have been applied to predict stop-bands for materials with both periodic and random geometrical microstructure, see for instance Liu et al. (2000) and Sigalas et al. (2005). In what follows, the analysis will be focused on compressional wave propagation in a two-phase bar and two techniques will be employed: the Plane-wave expansion method (Kushwaha et al. 1993; Sigalas and Economou 1994), and the Finite difference time domain method (Vasseur et al. 2001; Lu et al. 2009; Yukihiro et al. 2000).

**Fig. 2:**
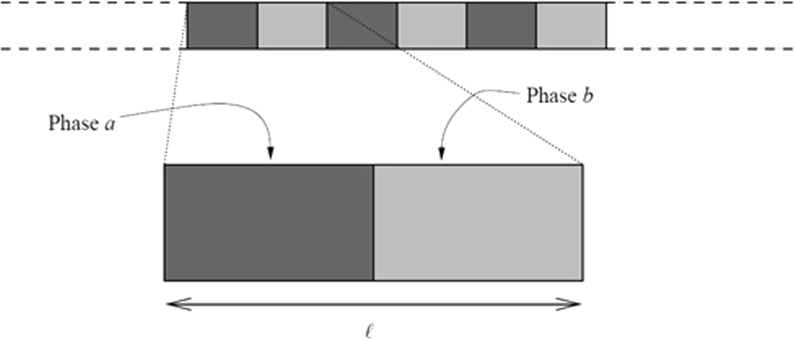
The laminate under study is a periodic two-phase material with unit cell length $\ell$ and equal amounts of each phase within the unit cell

The mass density and Young’s modulus of each phase comprising the unit cell, their volume fraction and the size of the unit cell relative to the medium itself are all parameters that influence the stop-band phenomenon. However a thorough study of their influence on the properties of stop-bands does not appear to exist in the literature. Furthermore, when these parameters are subject to various degrees of random perturbation it is not clear how this will affect the stop-band properties. Thus, in this paper randomness in both *mechanical* and *geometric* properties will be studied; the analyses will be carried out numerically and the wave filter effects will be compared with those of the undisturbed, periodic medium.

## Set-up of the numerical experiment

Figure 1 illustrates the configuration analysed throughout: numerical simulations, using the Newmark constant average acceleration time integration method, are conducted on a finite bar of total length *L*. This bar comprises four different regions $$V_0 , V_1 ,V_2 $$ and $$V_3 $$ with the last three being split up into subdomains $$V_j^l $$ and $$V_j^r $$ which are located on the left and right of the domain $$V_0 $$, respectively. In order to ensure that no waves are reflected back into the domain of interest, we have chosen a bar length with sufficiently large zones beyond the actual domain of interest. In order to slow down wave propagation significantly, two zones of impedance-matched layers have been taken on either side of the central zone, leading to $$L/L_0 =2.2$$ (see below for full details). The medium in $$V_1 $$ is chosen to have properties that correspond to the harmonic mean of the Young’s modulus and arithmetic mean of the density of the material that occupies $$V_0 $$ (this will be discussed in more detail below).

The source of longitudinal elastic waves is located at the centre of region $$V_1^l $$ and the receiver is placed at the centre of the region $$V_1^r $$. Regions $$V_2 $$ and $$V_3 $$ are so-called Perfectly Matched Layers (PMLs) and are impedance matched to $$V_1 $$. PMLs are used here as an alternative to absorbing boundary conditions. PMLs slow the wave down, ensuring that no reflections can be generated which would travel back into the domain of interest over the timescale of the simulation. In order to ensure equal impedance across regions $$V_1 , V_2 $$ and $$V_3 $$, we set $$\sqrt{\rho _1 E_1 }=\sqrt{\rho _2 E_2 }=\sqrt{\rho _3 E_3 }$$. The density and Young’s modulus contrasts in these domains are taken as follows: $$\rho _2 =10\rho _1 , \rho _3 =50\rho _1 $$ and $$E_2 =0.1E_1 , E_3 =0.02E_1 $$. This implies that the wave speeds $$c_1 , c_2 $$ and $$c_3 $$ in the outer sub-domains are related by the expressions $$c_2 =\sqrt{E_2 /\rho _2 }=0.1c_1 $$ and $$c_3 =\sqrt{E_3 /\rho _3 }=0.02c_1 $$, noting that the wave speeds in the PMLs are very small as required.

The microstructure of the material occupying sub-domain $$V_0 $$ is defined by a repeating unit cell comprising of two phases denoted by *a* and *b* with associated Young’s moduli and densities $$E_a , E_b $$ and $$\rho _a , \rho _b $$ respectively (see Fig. 2). For simplicity, equal volume fractions for both phases are assumed. Young’s modulus and density of phase *a* are taken as $$E_a =2\times 10^{11}\,\hbox {Pa}$$ and $$\rho _a =8\times 10^{3}\,\hbox {kg}/\hbox {m}^{3}$$, whereas the material properties of phase *b* are defined through contrast parameters $$\beta _E =E_b /E_a $$ and $$\beta _\rho =\rho _b /\rho _a $$. In order to identify the influences of relative Young’s moduli, densities and geometrical properties (in terms of unit cell lengths) on the band-gap structures, numerical analysis of longitudinal wave propagation through the finite domain occupied by the composite material will be performed, enabling the prediction of the associated transmission coefficient. Its magnitude will indicate the presence of either a stop-band or pass-band.

**Fig. 3:**
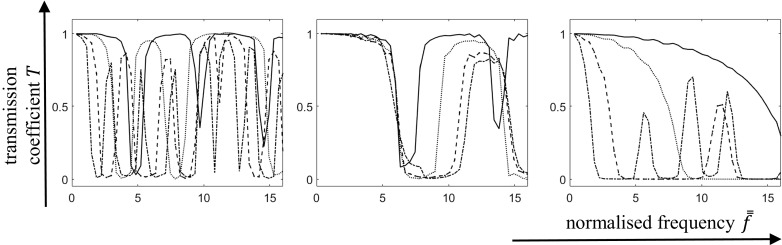
The transmission coefficient *T* as a function of normalised frequency $\bar{\bar{f}}$ for the given configuration: contrast in Young’s modulus of material’s phases (*left*); contrast in density of the material’s phases (*center*); contrast with respect to the overall length of unit cell lengths of the phases (*right*). Contrast parameters $\beta _E$ and $\beta _\rho$ range from 0.5—*solid*, 0.25—*dotted*, 0.1—*dashed*, and 0.05—*dot-dashed* (*left* and *center*); and unit cell length ranges from $0.002\hbox { m}$—*solid*, $0.004\hbox { m}$—*dotted*, $0.01\hbox { m}$–*dashed*, and $0.02\hbox { m}$—*dot-dashed* (*right*)

The transmission coefficient can be defined as $T(f)=\frac{A(f)}{B(f)}$, with amplitudes *A*(*f*) and *B*(*f*) being obtained after Fourier transform of a received displacement, following a continuous sine wave passing through homogeneous (resulting in *B*(*f*)) and heterogeneous (resulting in *A*(*f*)) specimens. The sine wave starts at $t=0$ with angular frequency $\omega$, amplitude ${{\mathcal {F}}}$ and associated forcing $F=\mathcal{F}cos\left( {\omega t} \right)$ at the source point and, as usual, frequency $f=\frac{\omega }{2\pi }.$

In a finite domain simulated numerically, it is expected that there may always be a very small amount of energy transmitted; thus a stop-band criterion is adopted according to which a frequency resides in a stop-band when $T\le 0.05$. In all tests an angular frequency ranging from $\omega =10^{5}\,\hbox {rad}/\hbox {s}$ to $4.5\times 10^{6}\,\hbox {rad}/\hbox {s}$, in intervals of $5\times 10^{4}\,\hbox {rad}/\hbox {s}$ is considered.

## Influence of mechanical and geometrical properties of a periodic composite

In this section the influence of deterministic mechanical and geometrical properties will be studied. Of specific interest are the contrasts in these properties between phases. These results are well known from the literature but serve as benchmarks for the analyses of non-periodicity reported in Sect. 4. Three different sets of parametric studies were carried out as follows:

- Contrast in Young’s moduli: Vary the contrast parameter $\beta _E$ whilst keeping $\beta _\rho =1$ and $\ell /L_0 =0.1$ Four different contrasts have been analysed: $\beta _E =0.05$, 0.1, 0.25, 0.5,
- Contrasts in mass densities: Vary the density contrast parameter $\beta _\rho$ whilst keeping $\beta _E =1$ and $\ell /L_0 =0.1$. Four different contrasts have been analysed: $\beta _\rho =0.05$, 0.1, 0.25, 0.5,
- Variation in unit cell lengths: Take $\ell =0.002\hbox { m}, \ell =0.004\,\hbox { m}, \ell =0.01\,\hbox { m}$ and $\ell =0.02\,\hbox { m}$, while keeping constant $\beta _E =0.25,\, \beta _\rho =0.1$ and $L_0 =0.1\,\hbox {m}$.

Predictions of the transmission coefficients associated with these three parametric studies are presented in Fig. 3 (left, center and right, respectively).

Note that transmission coefficients are presented here as functions of *normalised* frequencies. The normalisation has been performed with respect to the characteristic time scale $t_c =L_o /c$ (with averaged microstructural properties used in order to compute *c*) via $\bar{\bar{f}} =f*t_c$.

The results are summarised as follows:

- Increasing the contrast in Young’s moduli (decreasing $\beta _E )$, leads to a band-gap at lower frequency and the transmission coefficient in the pass-band drops slightly. Low frequency band-gap widths are relatively insensitive to changes in $\beta _\rho$ however (Fig. 3-left);
- Increasing the contrast in density leads to a significant increase in the width of the first stop-band and the transmission coefficient associated with the second pass-band also decreases (Fig. 3-centre);
- Increasing the unit cell length whilst keeping $$L_0 $$ fixed gives rise to a stop-band at lower frequency (Fig. 3-right).

## Influence of randomness on the band-gap structure of composites

So far the discussion has focussed on materials with heterogeneous but strictly periodic structure. In this section, the influence of randomness in the mechanical and geometrical parameters will be studied.

The reference (periodic) case with $E_a =2\times 10^{11}\,\hbox {Pa}$ and $\rho _a =8\times 10^{3}\,\hbox {kg}/\hbox {m}^{3}$ as defined in Sect. 2 and contrast parameters $\beta _E =0.25$ and $\beta _\rho =0.1$, unit cell length $\ell =0.01$ and test specimen length $L_0 =0.1\,\hbox {m}$ has been taken. A normal distribution with mean $\mu$ and increasing standard deviation ${\upsigma }$, resulting in coefficient of variation $C_v ={\upsigma }/\mu$ (see Table 1), has been assumed to represent the random character of corresponding parameters. Specific values of coefficients of variations for Young’s moduli, mass densities and unit cell lengths are indicated in Table 1. The top line of Table 1 contains the periodic reference case. For each case, five realisations have been taken.

**Table 1:** Randomness in mechanical and geometrical parameters: associated random properties

Case	$C_v \left( {E_a } \right)$	$C_v \left( {E_b } \right)$	$C_v \left( {\rho _a } \right)$	$C_v \left( {\rho _b } \right)$	$C_v \left( {l_a } \right)$	$C_v \left( {l_b } \right)$
Periodic	0	0	0	0	0	0
Random Young’s moduli	0.05	0.05	0	0	0	0
	0.1	0.1	0	0	0	0
	0.2	0.2	0	0	0	0
Random densities	0	0	0.05	0.05	0	0
	0	0	0.1	0.1	0	0
	0	0	0.2	0.2	0	0
Random geometry	0	0	0	0	0.05	0.05
	0	0	0	0	0.1	0.1
	0	0	0	0	0.2	0.2

**Fig. 4:**
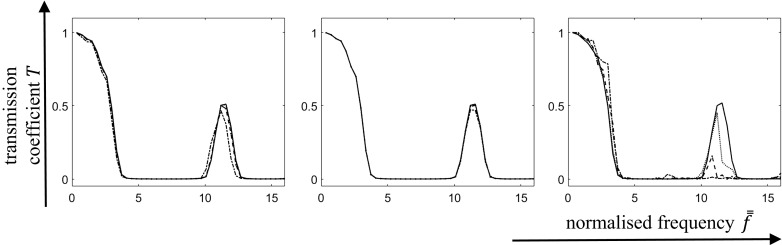
The (averaged) transmission coefficient *T* as a function of normalised frequency $\bar{\bar{f}}$ for the given configuration: randomness in Young’s modulus of material’s phases (*left*); randomness in density of the material’s phases (*center*); randomness in unit cell lengths of the phases (*right*). Coefficients of variation ranges from 0—*solid*, 0.05—*dotted*, 0.1—*dashed*, and 0.2—*dot-dashed*

In Fig. 4 the average transmission coefficients as functions of frequency are plotted for the cases of randomness introduced in Young’s moduli (Fig. 4-left), densities (Fig. 4-centre) and geometry (Fig. 4-right).

It is clear that randomness in both the Young’s moduli and density has a minimal effect on the band-gap structure of composites. The picture changes dramatically when randomness is introduced in the *geometry* of a material’s microstructure: as it can be seen in Fig. 4-right. In the second pass-band the transmission coefficient drops significantly with increasing contrast while increasing the coefficient of variation; this means that adding moderate perturbations to the geometry transforms an existing pass-band into a stop-band.

This can be understood as follows. From Fig. 3-right it is clear that the position of the first pass-band scales directly with the value of the unit cell length, and higher pass-bands appear at certain intervals along the frequency axis. However, when this is translated into corresponding wave lengths $\lambda$ according to $\lambda =c/f$ (taking the averaged material properties to compute *c*), it becomes clear that the higher pass-bands are associated with smaller wave lengths; these smaller wave lengths eventually become smaller than the length of the unit cell. Thus, a randomised unit cell length has very little influence on the position and extent of the first pass-band, but it affects the subsequent pass-sbands.

## Conclusions

In this study, the influence of both heterogeneous mechanical and geometrical properties on wave propagation has been tested, in particular their effects on stop-bands. Randomness in the mechanical properties does not appear to affect band-gap structure significantly. On the other hand, randomness in the geometrical properties, even in the form of moderate perturbations, can lead to a significant reduction of the transmission coefficient in the second pass-band, and, eventually, with sufficient randomness, this second pass-band can be transformed into a stop-band. This difference can be ascribed to the fact that in this study the source of heterogeneity is predominantly a geometrical distribution of material phases configured in series.
